# Supplementary material for: A systems approach discovers the role and characteristics of seven LysR type transcription factors in Escherichia coli
Source: Sci Rep. 2022 May 4;12:7274. doi: 10.1038/s41598-022-11134-7 (PMC9068703; doi:10.1038/s41598-022-11134-7)
Supplement: Supplementary file 1 — Supplementary Information 1. [file 41598_2022_11134_MOESM1_ESM.docx]

**Supplemental materials.**

RNA sequencing. Paired-end RNA-sequencing libraries were prepared as described (38).

Raw-sequencing reads were mapped to the reference genome (NC_000913.3) of *E. coli* K-12 MG1655 using bowtie (v1.1.2) with the following options “-X 1000 -n 2 -3 3”. Transcript abundance was quantified using *summarizeOverlaps* from the R *GenomicAlignments* package (v1.18.0) with the following options: “mode = “IntersectionStrict”, singleEnd = FALSE, ignore.strand = FALSE, preprocess.reads = invertStrand”. To ensure the quality of the compendium, genes shorter than 100 nucleotides and genes with under 10 fragments per million-mapped reads across all samples were removed before further analysis. Transcripts per million (TPM) were calculated by *DESeq2* (v1.22.1). The final expression values were log-transformed log^2^(TPM + 1), referred to as log-TPM.

**ChIP-Exo experiments.** Chip-exo experiments were performed following the procedures previously described. To identify PtrR binding sites for each strain, the DNA bound to PtrR from formaldehyde cross-linked cells, collected after growth in M9, was isolated by chromatin immunoprecipitation (ChIP) with the antibodies that specifically recognize the myc tag (9E10, Santa Cruz Biotechnology), and Dynabeads Pan Mouse IgG magnetic beads (Invitrogen) were added, followed by stringent washings as described previously ^2^. ChIP materials (chromatin-beads) were used to perform on-bead enzymatic reactions of the ChIP-exo method. Briefly, the sheared DNA of the chromatin-beads was repaired by the NEBNext End Repair Module (New England Biolabs), followed by the addition of a single dA overhang and ligation of the first adaptor (5’-phosphorylated) using a dA-Tailing Module (New England Biolabs) and NEBNext Quick Ligation Module (New England Biolabs), respectively. Nick repair was performed by using the PreCR Repair Mix (New England Biolabs). Lambda exonuclease- and RecJf exonuclease-treated chromatin was eluted from the beads, and overnight incubation at 65 degrees reversed the protein-DNA cross-links. RNA- and protein-free DNA samples were used to perform primer extension and second adaptor ligation with the following modifications. The DNA samples, incubated for primer extension as described previously (38), were treated with the dA-Tailing Module and NEBNext Quick Ligation Module (New England Biolabs) for second adaptor ligation. The DNA sample, purified using the GeneRead Size Selection Kit (Qiagen), was enriched by polymerase chain reaction (PCR) using Phusion High-Fidelity DNA Polymerase (New England Biolabs). The amplified DNA samples were purified again with a GeneRead Size Selection Kit (Qiagen) and quantified using Qubit dsDNA HS Assay Kit (Life Technologies). The quality of the DNA sample was checked by running the Agilent High Sensitivity DNA Kit using an Agilent 2100 Bioanalyzer before sequencing using HiSeq 2500 (Illumina) following the manufacturer’s instructions. Each modified step was also performed following the manufacturer’s instructions. ChIP-exo experiments were performed in duplicate.

**FIGURES**

**Fig. S1** Alignment of the LTTR transcription factors in Escherichia coli K-12 MG1655. Sequence profiles of the proteins were aligned in Clustal Omega. One of the most common regulator families in prokaryotes is lysR-type transcriptional regulators (LTTR). The lysR-type HTH domain is a DNA-binding, winged-turn-helix (wHTH) domain of about 60 residues present in the N-terminal part. LTTRs are present in diverse bacterial genera, archaea and algal chloroplasts. All LTTRs contain the DNA-binding lysR-type HTH domain.


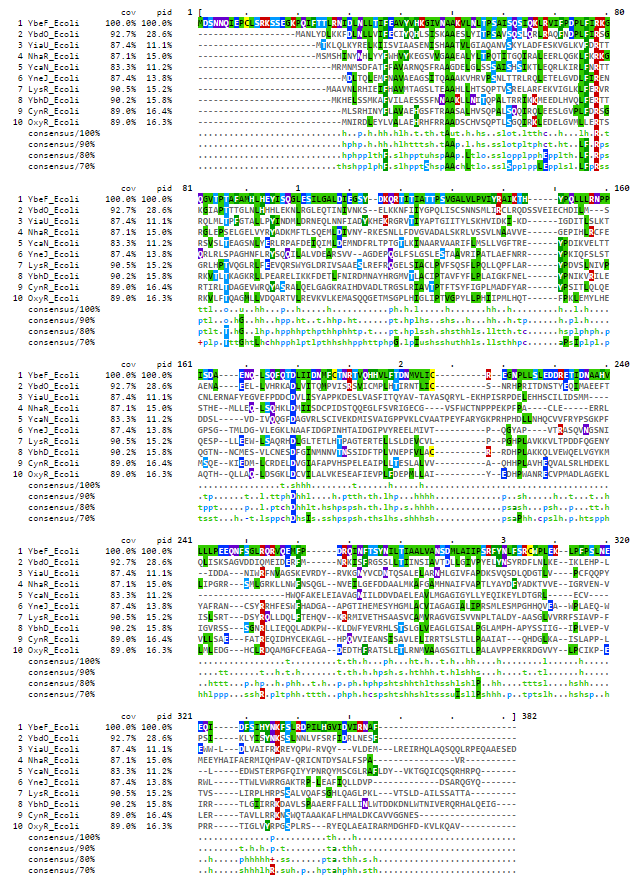


**Fig. S2** The predicted YbdO binding site (yellow backgrond) upstream of *ybdO* gene, suggesting that *ybdO* is autoregulator as supported by ChiP-exo DNA-binding and RNA-seq data (Table 2), the ybdO start of the gene is marked by red font.

| \| display  items per page \| \| --- \| |
| --- | --- |
| \| T \| displaying 1 - 10 of 11 \| next»  last» \| \| --- \| --- \| --- \| |
| \| **ID** \| **Organism** \| **Annotation** \| \| --- \| --- \| --- \| \| fig\|83333.1.peg.604 \| Escherichia coli K12 \| LysR-family transcriptional regulator YbdO \| \| fig\|1068608.3.peg.3106 \| Escherichia coli XH140A \| LysR-family transcriptional regulator YbdO \| \| fig\|457401.3.peg.1903 \| Escherichia sp. 4_1_40B \| Putative LysR-family transcriptional regulator YbdO \| \| fig\|556266.3.peg.3380 \| Shigella sp. D9 \| Putative LysR-family transcriptional regulator YbdO \| \| fig\|766158.3.peg.679 \| Shigella flexneri 2850-71 \| Putative LysR-family transcriptional regulator YbdO \| \| fig\|216599.1.peg.581 \| Shigella sonnei 53G \| Putative LysR-family transcriptional regulator YbdO \| \| fig\|621.8.peg.5123 \| Shigella boydii ATCC 9905 \| Putative LysR-family transcriptional regulator YbdO \| \| fig\|358708.5.peg.2095 \| Shigella dysenteriae 1012 \| Putative LysR-family transcriptional regulator YbdO \| \| fig\|754333.3.peg.2625 \| Escherichia albertii TW11588 \| Putative LysR-family transcriptional regulator YbdO \| \| fig\|585054.5.peg.2409 \| Escherichia fergusonii ATCC 35469 \| Putative LysR-family transcriptional regulator YbdO \|    |

Bottom of Form

fig|83333.1.peg.604 Escherichia coli K12

fig|1110693.3.peg.466 Escherichia coli str. K-12 substr. MDS42

fig|457401.3.peg.1903 Escherichia sp. 4_1_40B

fig|556266.3.peg.3380 Shigella sp. D9

fig|624.753.peg.3857 Shigella sonnei strain NLAE-zl-G496

fig|373384.11.peg.637 Shigella flexneri 5 str. 8401

fig|621.221.peg.5428 Shigella boydii strain ATCC 35964

fig|564.14.peg.4142 Escherichia fergusonii strain FDAARGOS_170

fig|622.435.peg.3171 Shigella dysenteriae strain ATCC 12039

fig|208962.68.peg.595 Escherichia albertii strain 1551-2

fig|1499973.6.peg.1976 Escherichia marmotae strain HT073016

fig|67829.4.peg.1024 Citrobacter murliniae strain P080C CL

fig|67827.10.peg.3160 Citrobacter werkmanii strain FDAARGOS_364

fig|1006003.3.peg.132 Citrobacter freundii ATCC 8090 = MTCC 1658

fig|545.11.peg.827 Citrobacter koseri strain FDAARGOS_86

fig|133448.4.peg.3409 Citrobacter youngae strain L6

fig|469595.3.peg.1261 Citrobacter sp. 30_2

fig|1218086.3.peg.1181 Citrobacter sedlakii NBRC 105722

fig|57706.65.peg.4328 Citrobacter braakii strain MiY-A

fig|1218085.3.peg.3193 Citrobacter rodentium NBRC 105723

fig|1064551.5.peg.662 Salmonella enterica subsp. enterica serovar Thompson str. RM6836

fig|208962.68.peg.595 t-agaaaaa**CTTAA**cgataaa**TTAAG**taaagaa-a-----------ataa

fig|1499973.6.peg.1976 t-agaaaaa**CTTAA**tcgtgaa**TTAAG**taaaata-acataatatataaaaa

fig|564.14.peg.4142 t-agaaaat**CTTAA**tccaaag**TTAAG**cagaaaa-acataat----acata

fig|622.435.peg.3171 t-agaaaaa**CTTAA**tcctgag**TTAAG**taaaaaa-acataat----ataaa

fig|373384.11.peg.637 t-agaaaaa**CTTAA**tcctgag**TTAAG**caaaaaa-acataatcaataaaaa

fig|621.221.peg.5428 t-agaaaaa**CTTAA**tcctgag**TTAAG**caaaaaa-acataatcaataaaaa

fig|556266.3.peg.3380 t-agaaaaa**CTTAA**tcctgag**TTAAG**caaaaaa-atataatcaataaaaa

fig|624.753.peg.3857 t-agaaaaa**CTTAA**tcctgag**TTAAG**caaaaaa-atataatcaataaaaa

fig|83333.1.peg.604 t-agaaaaa**CTTAA**tcctgag**TTAAG**caaaaaa-acataatcaataaaaa

fig|1110693.3.peg.466 t-agaaaaa**CTTAA**tcctgag**TTAAG**caaaaaa-acataatcaataaaaa

fig|457401.3.peg.1903 t-agaaaaa**CTTAA**tcctgag**TTAAG**caaaaaa--cataatcaataaaaa

fig|67829.4.peg.1024 t-agttttag**TTAA**ctgttca**TTAAG**atttaaatggatatgtgctaagaa

fig|67827.10.peg.3160 t-agtttaag**TTAA**ctattca**TTAAG**cgtataaaagattcttaaaagaaa

fig|1006003.3.peg.132 t-agctaaag**TTAA**ttattca**TTAAG**cacacaa-acataaatttcaagaa

fig|57706.65.peg.4328 ttagtttaaa**TTAA**taattca**TTAAG**cacacaa-acataaatcacaagaa

fig|133448.4.peg.3409 t-agtttaag**TTAA**ttattca**TTAAG**c-cataa-acataaactctaagaa

fig|469595.3.peg.1261 t-agtttaag**TTAA**ttattca**TTAAG**c-cataa-acataaactctaagaa

fig|545.11.peg.827 t-agtattat**T**g**AA**agagaat**TTAAG**cttagcaaacatcactattgtaaa

fig|1218086.3.peg.1181 t-agccata**CT**a**AA**tgtcact**TTAAG**tttg-cattgaaaaattttattca

fig|1218085.3.peg.3193 t-agccata**CT**a**AA**actaact**TTAAG**tcaggcattgatttctttagctta

fig|1064551.5.peg.662 t-agtttaaa**T**a**AA**ctctaca**TTAAG**tttaataaacataactctggtaaa

* ** * ** ***** * *

fig|1499973.6.peg.1976 tacataaagcca-tatcattgacttgatgaata--ttatgagaacaagca

fig|564.14.peg.4142 taaatacaacaa-cattattgaacaagtgaatgatttatggaagagacaa

fig|622.435.peg.3171 taca-acagcag-cattattgaacaaatgaatgatttatagaagagataa

fig|373384.11.peg.637 tacatacgcaaa-aaacattgattaagtgaatataccatggaagaaa-aa

fig|621.221.peg.5428 tacatacgcaaa-aaacattgattaagtgaatataccatggaagaaa-aa

fig|556266.3.peg.3380 tatatacgcaaa-aaacattgattaagtgaatataccatggaagaaa-aa

fig|624.753.peg.3857 tatatacgcaaa-aaacattgattaagtgaatataccatggaagaaa-aa

fig|83333.1.peg.604 tatatacgcaaa-aaacattgattaagtgaatatatcatggaagaaa-aa

fig|1110693.3.peg.466 tatatacgcaaa-aaacattgattaagtgaatatatcatggaagaaa-aa

fig|457401.3.peg.1903 tatatacgcaaa-aaacattgattaagtgaatatatcatggaagaaa-aa

fig|67829.4.peg.1024 tgattaataac--ttagaca--taatgaaacggaatatttcagagcaaag

fig|67827.10.peg.3160 taatttgcaat--tcaaata--tataaaaatgaaatatttccacataaaa

fig|1006003.3.peg.132 taactaccatt--taaacta--ttccacaattgaatttttcactacatta

fig|57706.65.peg.4328 tattcacaact--taaaata--tttcagaattaaatttttcaaaacatga

fig|133448.4.peg.3409 tgattagaatt--taaatta--ttcaacaattgaattatcctatacatta

fig|469595.3.peg.1261 tgattagaatt--taaatta--ttcaacaattgaattatcctatacatta

fig|545.11.peg.827 gcattggatgtg-ttgcataactataacaaatatagttatgacaatagtc

fig|208962.68.peg.595 tacataaagcca-tactattgacttgatgaata--ttatgataa-aagca

fig|1218086.3.peg.1181 gtgacaacacctaaaacattcatgcaacagataatttcatcttagaaa-t

fig|1218085.3.peg.3193 a-gatgacacggaaagcattaatgatacggatgatttcatatcaaaaagt

fig|1064551.5.peg.662 ttattgagatt--aagtcataat--catggaagataccatgataatgc--

fig|1499973.6.peg.1976 t-----atga-atggagtgcatcATGGCTAATCTTTACGACTTAAAAAAA

fig|564.14.peg.4142 t-----acaa--cggagtatgttATGGCTAATCTTTATGATCTAAAAAAA

fig|622.435.peg.3171 t-----atga--cggagtatgttATGGCGAATCTTTATGGCCTAAAAAAA

fig|373384.11.peg.637 t-----ataaaccggagtagtgtATGGCCAATCTCTACGACTTGAAAAAG

fig|621.221.peg.5428 t-----ataaaccggagtagtgtATGGCCAATCTCTACGACTTGAAAAAG

fig|556266.3.peg.3380 t-----ataa-ccggagtagtgtATGGCCAATCTCTACGACTTGAAAAAG

fig|624.753.peg.3857 t-----ataa-ccggagtagtgtATGGCCAATCTCTACGACTTGAAAAAG

fig|83333.1.peg.604 t-----ataa-ccggagtagtgtATGGCCAATCTCTACGACTTGAAAAAG

fig|1110693.3.peg.466 t-----ataa-ccggagtagtgtATGGCCAATCTCTACGACTTGAAAAAG

fig|457401.3.peg.1903 t-----ataa-ccggagtagtgtATGGCCAATCTCTACGACTTGAAAAAG

fig|67829.4.peg.1024 c-taagacaggaatggata---tATGGCTAACCTTTACGACCTCAAAAAA

fig|67827.10.peg.3160 t-aacaatgagaatataca---tATGGCTAACCTCTATGACCTTAAAAAA

fig|1006003.3.peg.132 c-catcacaggaaggtacg---cATGGCTAACCTCTATGACCTCAAAAAG

fig|57706.65.peg.4328 c-catcacaggaaggtatg---tATGGCTAACCTCTATGACCTTAAAAAG

fig|133448.4.peg.3409 c-catcacaggaaggtatg---cATGGCTAACCTCTATGACCTCAAAAAA

fig|469595.3.peg.1261 c-catcacaggaaggtatg---cATGGCTAACCTCTATGACCTCAAAAAA

fig|545.11.peg.827 a-caaaacaggaagaaaaaaa-cATGGCTAATCTCTACGATCTCAAAAAA

fig|208962.68.peg.595 tgaatcatga-atggagtgcgtcATGGCTAATCTGTATGATTTAAAGAAA

fig|1218086.3.peg.1181 g------gaaacaggaaaaaaacATGGCTAACCTTTACGATCTCAAAAAA

fig|1218085.3.peg.3193 g------agaaaaggaaaaacacATGGCTAACCTTTACGATCTCAAAAAG

fig|1064551.5.peg.662 -----------cgggaacatactATGGCTAACCTTTACGACCTTAAAAAA

***** ** ** ** * * ** **

**Fig S3** YbeF and ybdO gene clustering with citCDEFXGT (citrate lyase) and citAB(transcriptional regulator)in E. coli, Salmonella, Citrobacter sp.

**Fig. S4**

**Fig. S4** FlhDC regulon and additional flagella related genes form “FlhDC” iModulon (iModulonDB), transcriptional activity for yTF mutant strains in mid-exponential (WT, ybdO, ybeF, ygfI and late exponential (WT_LL, ycaN_LL, yiaU_LL) growth phase.

**Fig. S5A** *YbhJ* (red) genome context with the *ybhD* (dark purple), *ybhI* (dark blue), Gnl-2-oxoglutarate/malate translocator precursor and *ybhH* (dark salad green).

**
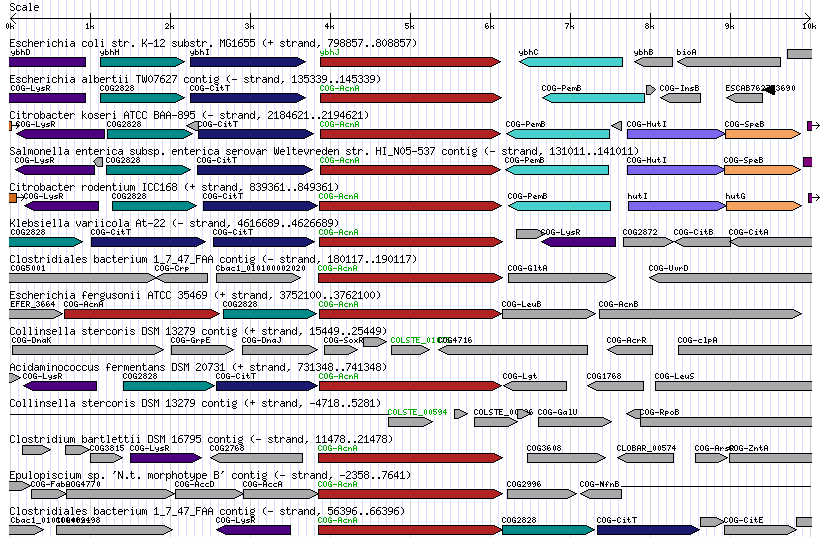
**

**Fig S5B YbhI alignment with the Gnl-2-oxoglutarate/malate translocator precursor from Spinach (TCDB).**

>gnl|BL_ORD_ID|13007 gnl|TC-DB|Q41364|2.A.47.3.1 2-OXOGLUTARATE/MALATE

TRANSLOCATOR PRECURSOR - Spinacia oleracea (Spinach). Length = 569

Score = 810, Bits(316), Expect = 9.11157e-101

Identities=35.7%, Positives=56.4%

Query: 5SLWKLILILAIPCIIGFMPAPAGLSELAWVLFGIYLAAIVGLVIKPFPEPVVLLIAVAAS 64

S+ L+ + I I F+ P P G+S AW L I+ L+ IVG++ +P P V L+ + AS

Sbjct:103SIKPLLASILTGVIIWFIPTPEGVSRNAWQLLAIFLSTIVGIITQPLPLGAVALMGLGAS162

Query: 65 MVVVGNLSDGAFKTTAVLSGYSSGTTWLVFSAFTLSAAFVTTGLGKRIAYLLIGKIGNTT 124

++ AF S + WL+ AF + F+ TGLG RIAY + G+++

Sbjct: 163 VLTKTLTFSAAF------SAFGDPIPWLIALAFFFARGFIKTGLGNRIAYQFVKLFGSSS 216

Query: 125 LGLGYVTVFLDLVLAPATPSNTARAGGIVLPIINSVAVALGSE-PEKSPRRVGHYLMMSI 183

LGLGY VF + +. LAPA PS +. ARAGGI LP++ S+ +. A GS + + R++G +. LM++

Sbjct: 217 LGLGYSLVFSEALLAPAIPSVSARAGGIFLPLVKSLCIACGSNVGDGTERKLGAWLMLTC 276

Query: 184 YMVTKTTSYMFFTAMAGNILALKMINDILHLQISWGGWALAAGLPGIIMLLVTPLVIYTM 243

+ + +. S MF TAMA N L+ + + + I W WA AA +. PG++ L+ V PL++ Y +

Sbjct: 277 FQTSVISSSMFLTAMAANPLSATLTFNTIGKAIGWMDWAKAAFVPGLVSLIVVPLLLYVV 336

Query: 244 YPPEIK-KVDNKTIAKAGLAELGPMKIREKMLLGVFVLALLGWIFSKSLGVDESTVAIVV 302

YPPEIK D + AK L ++. GPM E ++ +. L + W+F LGVD T AI+

Sbjct: 337 YPPEIKSSPDAPRLAKEKLDKMGPMTKNESIMAVTLLLTVGLWVFGGKLGVDAVTAAILG 396

Query: 303 MATMLLLGIVTWEDVVKNKGGWNTLIWYGGIIGLSSLLSKVKFFEWLAE-VFKNNLAFDG 361

++ +L+ G+VTW++ + W+TL W+ +I ++ L+K W +E V K

Sbjct: 397 LSVLLITGVVTWKECLAESVAWDTLTWFAALIAMAGYLNKYGLITWFSENVVKVVGGLGL 456

Query: 362 HGNVAFFVIIFLSIIVRYFFASGSAYIVAMLPVFAMLANVSGAPLMLTALALLFSNSYGG 421

++F V++ L YFFASG+A+I AM F +A+ G P L A+ L F ++ G

Sbjct: 457 SWQMSFGVLVLLYFYSHYFFASGAAHIGAMFTAFLSVASALGTPPFLAAIVLSFLSNLMG 516

Query: 422 MVTHYGGAAGPVIFGVGYNDIKSWWLVGAVLTILTFLVHITLGVWWWNMLIGW 474

+THYG + PV. +G Y + WW G +++I+ ++ + +G WW + W

Sbjct: 517 GLTHYGIGSAPVFYGANYVPLPQWWGYGFLISIVNLIIWLGVGGLWWKAIGLW 569

**Fig. S6 The** growth measurement of the *ybhD* mutant  (orange and grey line) compared to the wild type BW25113 strain of *E. coli* (blue and yellow line). Growth was measured in 96-well plates with glucose or glycerol as the carbon source (in the presence and the absence of 10 mM malate).

M9 medium, 0.4% glycerol carbon source, L-malate 10 mM

**Fig. S7** The microaerobic growth for the ygfI deletion mutant (blue,orange) and E. coli BW25113 (red, yellow) in M9 glucose medium (A) and the same medium supplemented with 7 mM L-threonine.

**Fig. S8** The predicted YneJ DNA-binding site (red font) upstream of *yneJ* gene, ChIP-Exo detected DNA is marked with green background**.**

**yneI<-**

fig|457401.3.peg.516 CCGGAGTAATGGT**CAT**cgggg----tatctcctttatgagtcatggtatgaagatacgcagatttactct

fig|1050617.5.peg.1967 CCGGAGTAATGGT**CAT**cgggg----tatctcctttatgagtcatggtatgaagatacgcagatttactct

fig|83333.1.peg.1513 CCGGAGTAATGGT**CAT**cgggg----tatctcctttatgagtcatggtatgaagatacgcagatttactct

fig|556266.3.peg.269 CCGGAGTAATGGT**CAT**cgggg----tatctcctttatgagtcatggtatgaagatacgcagatttactct

fig|344609.11.peg.1857 CCGGAGTAATGGT**CAT**cgggg----tatctcctttatgagtcatggtatgaagatacgcagatttactct

fig|216599.12.peg.1800 CCGGAGTAATGGT**CAT**cgggg----tatctcctttatgagtcatggtatgaagatacgcagatttactct

fig|300267.13.peg.1966 CCGGAGTAATGGT**CAT**cgggg----tatctcctttatgagtcatggtatgaagatacgcagatttactct

fig|564.14.peg.3012 CCGGAGTAATGGT**CAT**cgggg----tatctcctttatgagtcatggtatgaagatacgcagatttactct

fig|1499973.6.peg.993 CCGGAGTGATGGT**CAT**cgggg----tatctccttcatgagtcagggtaaaaagatacgcagatttactct

fig|208962.36.peg.1540 CCGGAGTGATGGA**CAT**cagga----tatctccattgtgagtgatatctccaggatacgcagatttactcc

fig|1006003.3.peg.577 CTGCTGTGATAGT**CAT**taagtgc-ctctctttatcgcagggtatggttatatcatgagcatatttactct

fig|1173691.3.peg.4273 CTGCTGTGATAGT**CAT**taagtgc-ctctctttatcgcagggtatggttatatcatgagcatatttactct

fig|67829.4.peg.3175 CTGCTGTGATGGT**CAT**aggtcttactctctttatcgcaggatatggttatatcatgagcgtatttactct

fig|545.23.peg.4274 CAGGTGTCATCGT**CAT**cggtttgtctctctacgttgcggga-atgcctgcataatgggcgcatttactct

fig|35703.30.peg.4088 CCGGTGAAATCGT**CAT**acag-----tctctccgtcgttcttgatgtcatcatcatggacgcgtttactct

* * * ** * *** * *** * * * ** * *******

YneJ binding area YneJ binding site **-> yneJ**

fig|457401.3.peg.516 tgctttaaaatgaataatattaagccactta**ttcacgaatcgagaa**tgct**ATG**GATCTGACCCAACTGGAGATGTT

fig|1050617.5.peg1967tgctttaaaatgaataatattaagccactta**ttcacgaatcgagaa**tgct**ATG**GATCTGACCCAACTGGAGATGTT

fig|83333.1.peg.1513 tgctttaaaatgaataatattaagccactta**ttcacgaatcgagaa**tgct**ATG**GATCTGACCCAACTGGAGATGTT

fig|556266.3.peg.269 tgctttaaaatgaataatattaagccactta**ttcacgaatcgagaa**tatt**ATG**GATCTGACCCAACTGGAGATGTT

fig|344609.11peg.1857tgctttaaaatgaataatattaagccactta**ttcacgaatcgagaa**tatt**ATG**GATCTGACCCAACTGGAGATGTT

fig|216599.12peg.1800tgctttaaaatgaataatattaagccactta**ttcacgaatcgagaa**tgtt**ATG**GATCTGACCCAACTGGAGATGTT

fig|300267.13.peg1966tgctttaaaatgaataatattaagccactta**ttcacaaatcgagaa**tatt**ATG**GATCTGACCCAACTGGAGATGTT

fig|564.14.peg.3012 tgctttaaaatgaataatattaagccactta**ttcacgaatcgagaa**tgtt**ATG**GATCTGACCCAACTGGAGATGTT

fig|1499973.6.peg.993tactttaaaatgaataatattaagccactta**ttcacgaattgagaa**tact**ATG**GATCTAACCCAACTGGAGATGTT

fig|208962.36.peg1540ttcgtaaaaatgaataatattaagtcactta**ttcacaaatcgagaa**tgcc**ATG**GACCTGACCCAGTTAGAGATGTT

fig|1006003.3.peg.577ttctgaaaaatgaataatattaaccatacca**ttcacgaatagagaa**aatt**ATG**GATCTGACGCAGCTGGAAATGTT

fig|1173691.3.peg4273ttctgaaaaatgaataatattaaccatacca**ttcacgaatagagaa**aatt**ATG**GATCTGACGCAGCTGGAAATGTT

fig|67829.4.peg.3175 ttatgaaaaatgaataatattaaccacacca**ttcacgaatagagaa**aaca**ATG**GATCTGACGCAACTGGAAATGTT

fig|545.23.peg.4274 ttctgaaaaatgaataatattaagcactgcg**ttcacaaaaagagaa**aatc**ATG**GATCTGACTCAGCTGGAAATGTT

fig|35703.30.peg.4088ttccgaaaaatgaataatattaaccaaacca**ttcacgaagagagaa**aacc**ATG**GATCTGACCCAACTGGAGATGTT

* ***************** ***** ** ***** **** ** ** ** * ** ****

**2^nd^ branch of YneJ in Serratia / Yersinia / Erwinia**

fig|1206776.4.peg.1570catgccgcagtattaaa-**ctcacaaaaaatgaa**taatactcatcaagttc**atcacgaaaagagaa**gac-cATGGA

fig|399741.3.peg.1566 catgccgcagtattaaa-**ctcacaaaaactgaa**taatactcaccaagttc**atcacgaaaagagaa**gac-aATGGA

fig|615.1.peg.4875 catgccgcagtattaaa-**ctcacaaaaactgaa**taatagttatcaagttc**atcacgaaacgagaa**tat-tATGGA

fig|349967.3.peg.2685 catgcctgagcattaga-**atcacaaaaaatgaa**taatagtcactaagttc**atcacaaaaagagaa**cacttATGGA

fig|349968.3.peg.1236 catgcctgagcattaga-**atcacaaaaaatgaa**taatactcattaagttc**atcacaaaaagagaa**tacatATGGA

fig|349966.3.peg.2345 catgcctgagcattaga-**atcacaaaaaatgaa**taattctcactaagttc**atcacaaaaagagaa**cgattATGGA

fig|349965.3.peg.1395 catgcctgagcattaga-**atcacaaaaaatgaa**taatgctcattaagttc**atcacaaaaagagaa**cacctATGGA

fig|630.2.peg.2749 catgcctgagcattaga-**atcacaaaaaatgaa**taatactcactaagttc**atcacggaaagagaA**TGCTTATGGA

fig|187410.1.peg.2620 catgcctgagtattaga-**atcacaaaaaatgaa**taatagtcattaagttc**atcacaaaaagagaa**catctATGGA

fig|273123.1.peg.1629 catgcctgagtattaga-**atcacaaaaaatgaa**taatagtcattaagttc**atcacaaaaagagaa**catctATGGA

fig|218491.3.peg.2039 cacagcttgccgctacac**ttcacaaaaaatgaa**taatcaaacaccagttc**attacgaaaagagaa**aac-cATGGA

** * ** * ********* ******** ******* ** ** **** *****

**-> yneJ**

**Fig.S9 A.** The aerobic growth for the ptrR (yneJ) (dark blue line) deletion mutant and *E. coli* BW25113 (orange line) in modified M9 medium glycerol (0.1%) as carbon source and 20 mM L-Glutamate (nitrogen source), and in 96-well plates and the same medium (with glycerol 0.4%) in culture tubes.

**B.** The phenotype in plates PM2 for the ptrR (yneJ) deletion mutant and *E. coli* BW25113 in M9 medium without carbon source, supplemented by L-glutamate (20mM)

**Fig. S10** The antibiotic resistance phenotype for the *ptrR* (*yneJ*) deletion mutant and *E. coli* BW25113 wild type (blue line) for the plate PM11C, Biolog for two different concentrations of tetracycline is **A.** 1X and **B.** 8X.
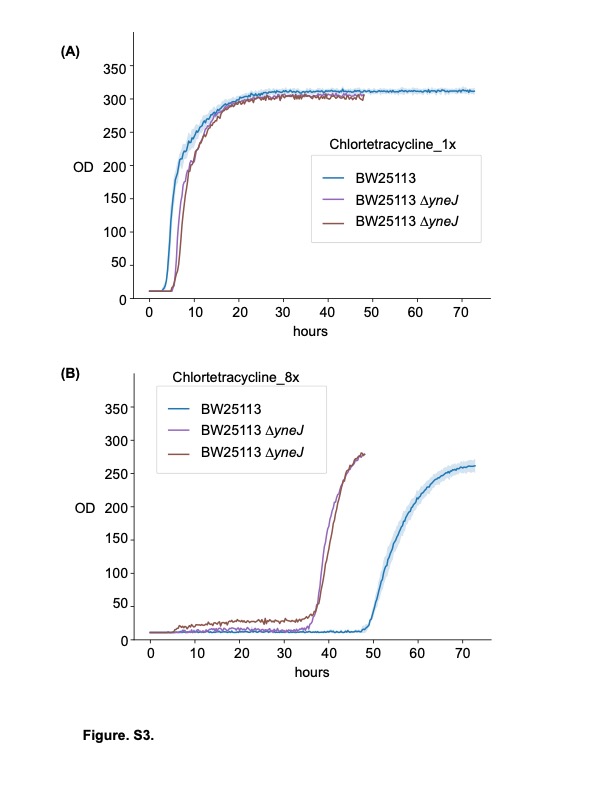


**TABLES**

**Table S1**

| b-number | *gene* | | Sequence | Position | Score | Log^2^ fold change |
| --- | --- | --- | --- | --- | --- | --- |
| b2724 | *hycB* | | GccGGACAAAGAACTC | -447 | 5.65 | -9.0 |
| \| b0903 \| \| --- \| | *pflB* | | GATGGACAAAGCGTTC | -356 | 7.26 | -5.39 |
| b1200 | *dhaK* | | GATGCACAAtGGATTC | -92 | 6.22 | -1.39 |
| b1241 | *adhB* | | GATGCtgAAAGGTGTC | -274 | 5.16 | -5.70 |
| b2503 | *pdeF* | GActCACAAAaGGTTC | | -365 | 5.16 | - |
| b4076 | *nrfA* | GATGaAtAAAGGGCTt | | -390 | 5.37 | - |
| b4020 | *yjbB* | GATtCACAAAtCTGTC | | -230 | 5.36 | - |
| b4028 | *yjbG* | GcTGGACgAAGAAGTC | | -153 | 4.80 | - |
| b1468 | *narZ* | GATGtACAAccGTGTC | | -75 | 5.87 | -2.28 |

**Table S2**
